# Supplementary material for: Hot spot prediction in protein-protein interactions by an ensemble system
Source: BMC Syst Biol. 2018 Dec 31;12(Suppl 9):132. doi: 10.1186/s12918-018-0665-8 (PMC6311905; doi:10.1186/s12918-018-0665-8)
Supplement: Supplementary file 2 — All relevant MATLAB code. A simple Matlab implement of our predictor. (ZIP 19,742 kb) [file 12918_2018_665_MOESM2_ESM.zip › Additional file 2/Read ASA/ASA website/dbMPIKT/3SE8H.html]

 NetSurfP - Protein Surface Accessibility and Secondary Structure Predictions

  

|  |  |  |
| --- | --- | --- |
|  |  | NetSurfP - Protein Surface Accessibility and Secondary Structure Predictions  Technical University of Denmark |

  


---

```
# For publication of results, please cite:
# A generic method for assignment of reliability scores applied to solvent accessibility predictions.
# Bent Petersen, Thomas Nordahl Petersen, Pernille Andersen, Morten Nielsen and Claus Lundegaard
# BMC Structural Biology 2009, 9:51 doi:10.1186/1472-6807-9-51
#
# Column 1: Class assignment - B for buried or E for Exposed - Threshold: 25% exposure, but not based on RSA
# Column 2: Amino acid
# Column 3: Sequence name
# Column 4: Amino acid number
# Column 5: Relative Surface Accessibility - RSA
# Column 6: Absolute Surface Accessibility
# Column 7: Z-fit score for RSA prediction
# Column 8: Probability for Alpha-Helix
# Column 9: Probability for Beta-strand
# Column 10: Probability for Coil
E Q  3SE8H                  1    0.754 134.664   1.150   0.003   0.003   0.994
B V  3SE8H                  2    0.238  36.519  -0.289   0.004   0.616   0.381
E Q  3SE8H                  3    0.435  77.709   1.340   0.001   0.900   0.099
B L  3SE8H                  4    0.157  28.838   0.428   0.001   0.900   0.099
E V  3SE8H                  5    0.419  64.431   0.507   0.003   0.718   0.279
B Q  3SE8H                  6    0.221  39.506   0.756   0.004   0.514   0.481
E S  3SE8H                  7    0.431  50.548  -0.296   0.004   0.138   0.858
E G  3SE8H                  8    0.526  41.380  -1.910   0.018   0.047   0.935
E A  3SE8H                  9    0.520  57.249  -2.361   0.018   0.047   0.935
E V  3SE8H                 10    0.524  80.523  -1.799   0.020   0.205   0.775
E I  3SE8H                 11    0.421  77.866  -1.141   0.022   0.359   0.619
E K  3SE8H                 12    0.328  67.449   0.554   0.021   0.451   0.528
E T  3SE8H                 13    0.462  64.024   1.090   0.020   0.205   0.775
E P  3SE8H                 14    0.461  65.359  -0.589   0.005   0.045   0.951
E G  3SE8H                 15    0.597  47.008  -1.829   0.005   0.045   0.951
E S  3SE8H                 16    0.429  50.232  -0.418   0.004   0.138   0.858
E S  3SE8H                 17    0.395  46.294  -0.252   0.004   0.616   0.381
B V  3SE8H                 18    0.138  21.149   0.798   0.001   0.900   0.099
E K  3SE8H                 19    0.383  78.721   0.831   0.001   0.900   0.099
B I  3SE8H                 20    0.074  13.709   0.372   0.001   0.959   0.040
B S  3SE8H                 21    0.152  17.768   0.696   0.001   0.900   0.099
B C  3SE8H                 22    0.037   5.251  -0.178   0.001   0.959   0.040
B R  3SE8H                 23    0.296  67.761   0.538   0.001   0.900   0.099
B A  3SE8H                 24    0.140  15.417  -0.499   0.002   0.816   0.182
B S  3SE8H                 25    0.195  22.877  -0.739   0.004   0.514   0.481
E G  3SE8H                 26    0.266  20.934  -1.841   0.004   0.197   0.799
B Y  3SE8H                 27    0.285  60.990  -1.246   0.005   0.262   0.733
B N  3SE8H                 28    0.222  32.559  -1.049   0.004   0.420   0.576
B F  3SE8H                 29    0.131  26.272  -0.802   0.021   0.451   0.528
B R  3SE8H                 30    0.269  61.555  -1.230   0.021   0.279   0.699
B D  3SE8H                 31    0.310  44.628  -1.216   0.018   0.088   0.893
B Y  3SE8H                 32    0.126  27.012   0.455   0.020   0.205   0.775
B S  3SE8H                 33    0.092  10.724   0.155   0.022   0.552   0.426
B I  3SE8H                 34    0.029   5.421   0.316   0.018   0.846   0.136
B H  3SE8H                 35    0.058  10.532   0.027   0.018   0.846   0.136
B W  3SE8H                 36    0.038   9.067   0.312   0.018   0.846   0.136
B V  3SE8H                 37    0.042   6.486   0.482   0.018   0.846   0.136
B R  3SE8H                 38    0.151  34.487   0.612   0.021   0.756   0.223
B L  3SE8H                 39    0.103  18.933   0.362   0.021   0.451   0.528
E I  3SE8H                 40    0.300  55.518   1.068   0.005   0.045   0.951
E P  3SE8H                 41    0.390  55.355   0.624   0.005   0.015   0.979
E D  3SE8H                 42    0.704 101.475  -1.952   0.005   0.015   0.979
E K  3SE8H                 43    0.432  88.801   0.486   0.018   0.019   0.964
E G  3SE8H                 44    0.477  37.516  -0.584   0.018   0.047   0.935
B F  3SE8H                 45    0.183  36.648   0.844   0.004   0.197   0.799
B E  3SE8H                 46    0.128  22.292  -0.338   0.004   0.616   0.381
B W  3SE8H                 47    0.052  12.578   0.229   0.001   0.900   0.099
B I  3SE8H                 48    0.023   4.292   0.926   0.001   0.959   0.040
B G  3SE8H                 49    0.019   1.487  -0.290   0.001   0.959   0.040
B W  3SE8H                 50    0.075  18.038  -0.240   0.001   0.959   0.040
B I  3SE8H                 51    0.021   3.885   0.948   0.001   0.959   0.040
B K  3SE8H                 52    0.158  32.480   0.517   0.002   0.816   0.182
B P  3SE8H                 53    0.219  31.019  -0.879   0.005   0.336   0.660
E L  3SE8H                 54    0.562 102.957  -1.597   0.005   0.015   0.979
E W  3SE8H                 55    0.567 136.412  -2.296   0.003   0.003   0.994
E G  3SE8H                 56    0.548  43.120  -2.429   0.018   0.019   0.964
E A  3SE8H                 57    0.478  52.709  -0.582   0.018   0.047   0.935
E V  3SE8H                 58    0.329  50.613  -0.227   0.020   0.205   0.775
E S  3SE8H                 59    0.429  50.255   0.092   0.021   0.279   0.699
B Y  3SE8H                 60    0.228  48.788   0.375   0.020   0.205   0.775
B A  3SE8H                 61    0.210  23.142  -0.617   0.018   0.088   0.893
E R  3SE8H                 62    0.684 156.613  -0.460   0.018   0.047   0.935
E Q  3SE8H                 63    0.477  85.228  -0.176   0.018   0.047   0.935
B L  3SE8H                 64    0.086  15.765  -0.032   0.018   0.088   0.893
E Q  3SE8H                 65    0.458  81.710   1.007   0.018   0.088   0.893
B G  3SE8H                 66    0.232  18.282  -1.782   0.005   0.262   0.733
B R  3SE8H                 67    0.209  47.838   0.590   0.003   0.718   0.279
B V  3SE8H                 68    0.047   7.255  -0.242   0.001   0.900   0.099
B S  3SE8H                 69    0.109  12.810   0.417   0.001   0.900   0.099
B M  3SE8H                 70    0.043   8.604   0.402   0.001   0.900   0.099
B T  3SE8H                 71    0.064   8.807  -0.097   0.002   0.816   0.182
B R  3SE8H                 72    0.192  43.899   0.655   0.023   0.655   0.322
B Q  3SE8H                 73    0.142  25.397  -0.668   0.021   0.451   0.528
E L  3SE8H                 74    0.287  52.550  -0.267   0.056   0.142   0.802
B S  3SE8H                 75    0.134  15.705  -2.209   0.056   0.142   0.802
E Q  3SE8H                 76    0.450  80.441   0.428   0.056   0.142   0.802
E D  3SE8H                 77    0.340  48.965  -1.453   0.056   0.142   0.802
E P  3SE8H                 78    0.462  65.544  -1.045   0.056   0.142   0.802
E D  3SE8H                 79    0.476  68.563  -1.946   0.056   0.142   0.802
E D  3SE8H                 80    0.540  77.886  -1.686   0.056   0.142   0.802
E P  3SE8H                 81    0.526  74.611  -1.285   0.020   0.205   0.775
E D  3SE8H                 82    0.360  51.833  -1.367   0.020   0.205   0.775
E W  3SE8H                 83    0.334  80.279  -0.583   0.022   0.359   0.619
B G  3SE8H                 84    0.168  13.229  -0.805   0.004   0.514   0.481
B V  3SE8H                 85    0.099  15.155   0.270   0.002   0.816   0.182
B A  3SE8H                 86    0.038   4.243  -0.293   0.011   0.918   0.071
B Y  3SE8H                 87    0.112  23.956   0.308   0.001   0.959   0.040
B M  3SE8H                 88    0.035   7.004   0.222   0.001   0.959   0.040
B E  3SE8H                 89    0.157  27.445  -0.338   0.001   0.900   0.099
B F  3SE8H                 90    0.065  12.985  -0.438   0.023   0.655   0.322
E S  3SE8H                 91    0.409  47.970  -0.091   0.022   0.359   0.619
E G  3SE8H                 92    0.436  34.337  -0.638   0.019   0.141   0.840
B L  3SE8H                 93    0.132  24.133  -0.137   0.018   0.088   0.893
E T  3SE8H                 94    0.486  67.478   1.079   0.018   0.088   0.893
E P  3SE8H                 95    0.371  52.645  -0.492   0.018   0.088   0.893
E A  3SE8H                 96    0.515  56.764  -0.612   0.052   0.084   0.864
B D  3SE8H                 97    0.226  32.523  -0.691   0.052   0.084   0.864
B T  3SE8H                 98    0.160  22.206   0.240   0.069   0.386   0.545
B A  3SE8H                 99    0.040   4.430  -0.263   0.021   0.756   0.223
B E  3SE8H                100    0.092  16.003   1.050   0.018   0.846   0.136
B Y  3SE8H                101    0.038   8.121   0.708   0.018   0.846   0.136
B F  3SE8H                102    0.062  12.443   0.931   0.021   0.756   0.223
B C  3SE8H                103    0.016   2.190   1.458   0.023   0.655   0.322
B V  3SE8H                104    0.042   6.455  -0.195   0.022   0.552   0.426
B R  3SE8H                105    0.175  40.052   0.157   0.022   0.359   0.619
E R  3SE8H                106    0.385  88.142  -0.596   0.018   0.088   0.893
E G  3SE8H                107    0.340  26.782  -1.166   0.018   0.047   0.935
E S  3SE8H                108    0.463  54.275  -2.128   0.018   0.088   0.893
E C  3SE8H                109    0.426  59.881  -2.200   0.019   0.141   0.840
E D  3SE8H                110    0.413  59.470  -1.925   0.018   0.088   0.893
E Y  3SE8H                111    0.316  67.465  -2.539   0.018   0.088   0.893
B C  3SE8H                112    0.265  37.136  -1.930   0.018   0.088   0.893
E G  3SE8H                113    0.328  25.806  -1.946   0.018   0.088   0.893
E D  3SE8H                114    0.356  51.328  -1.681   0.018   0.047   0.935
B F  3SE8H                115    0.282  56.698  -0.931   0.018   0.088   0.893
B P  3SE8H                116    0.095  13.480  -0.558   0.019   0.141   0.840
B W  3SE8H                117    0.053  12.674  -0.169   0.020   0.205   0.775
B Q  3SE8H                118    0.074  13.216  -0.681   0.021   0.279   0.699
B Y  3SE8H                119    0.047  10.151  -0.313   0.022   0.359   0.619
B W  3SE8H                120    0.035   8.442  -0.615   0.020   0.205   0.775
B C  3SE8H                121    0.027   3.833  -1.573   0.020   0.205   0.775
B Q  3SE8H                122    0.117  20.986  -0.146   0.019   0.141   0.840
B G  3SE8H                123    0.066   5.163  -1.701   0.021   0.279   0.699
B T  3SE8H                124    0.059   8.142  -0.264   0.022   0.552   0.426
B V  3SE8H                125    0.164  25.222   0.040   0.021   0.756   0.223
B V  3SE8H                126    0.034   5.272  -0.716   0.023   0.655   0.322
B V  3SE8H                127    0.212  32.538  -0.396   0.022   0.552   0.426
B V  3SE8H                128    0.153  23.593  -1.028   0.021   0.451   0.528
B S  3SE8H                129    0.224  26.253  -0.971   0.005   0.262   0.733
E S  3SE8H                130    0.433  50.806  -1.457   0.019   0.141   0.840
E A  3SE8H                131    0.561  61.789  -0.948   0.018   0.088   0.893
E S  3SE8H                132    0.432  50.654  -0.890   0.053   0.043   0.903
E T  3SE8H                133    0.443  61.513  -1.127   0.018   0.047   0.935
E K  3SE8H                134    0.475  97.666  -1.523   0.018   0.047   0.935
E G  3SE8H                135    0.437  34.360  -1.235   0.018   0.047   0.935
E P  3SE8H                136    0.473  67.133  -1.703   0.018   0.019   0.964
E S  3SE8H                137    0.488  57.240  -1.592   0.018   0.047   0.935
E V  3SE8H                138    0.380  58.391  -0.730   0.019   0.141   0.840
B F  3SE8H                139    0.129  25.971  -0.171   0.020   0.205   0.775
E P  3SE8H                140    0.433  61.499  -1.060   0.021   0.279   0.699
B L  3SE8H                141    0.217  39.769  -1.855   0.004   0.197   0.799
B A  3SE8H                142    0.179  19.770  -2.099   0.005   0.045   0.951
B P  3SE8H                143    0.251  35.560  -2.355   0.005   0.015   0.979
E S  3SE8H                144    0.278  32.570  -1.903   0.005   0.015   0.979
E S  3SE8H                145    0.348  40.797  -2.434   0.005   0.015   0.979
E K  3SE8H                146    0.614 126.362  -2.483   0.005   0.015   0.979
E S  3SE8H                147    0.338  39.649  -2.029   0.005   0.015   0.979
E T  3SE8H                148    0.500  69.295  -1.468   0.003   0.003   0.994
E S  3SE8H                149    0.556  65.116  -1.418   0.003   0.003   0.994
E G  3SE8H                150    0.696  54.767  -1.809   0.003   0.003   0.994
E G  3SE8H                151    0.567  44.591  -1.693   0.005   0.045   0.951
E T  3SE8H                152    0.435  60.362   0.804   0.005   0.336   0.660
B A  3SE8H                153    0.078   8.540   0.403   0.001   0.900   0.099
B A  3SE8H                154    0.200  21.996   0.338   0.001   0.959   0.040
B L  3SE8H                155    0.046   8.496   0.383   0.001   0.959   0.040
B G  3SE8H                156    0.058   4.549   0.074   0.001   0.959   0.040
B C  3SE8H                157    0.025   3.510   0.582   0.001   0.900   0.099
B L  3SE8H                158    0.153  27.941   0.411   0.001   0.900   0.099
B V  3SE8H                159    0.054   8.377  -0.527   0.003   0.718   0.279
E K  3SE8H                160    0.322  66.338  -0.377   0.005   0.336   0.660
E D  3SE8H                161    0.513  73.981  -1.807   0.004   0.138   0.858
B Y  3SE8H                162    0.225  48.147  -0.802   0.004   0.138   0.858
E F  3SE8H                163    0.396  79.437  -0.410   0.004   0.197   0.799
B P  3SE8H                164    0.237  33.588  -1.743   0.004   0.138   0.858
E E  3SE8H                165    0.574 100.330  -1.214   0.004   0.085   0.910
E P  3SE8H                166    0.416  59.073  -1.687   0.004   0.197   0.799
B V  3SE8H                167    0.166  25.453  -0.179   0.003   0.718   0.279
E T  3SE8H                168    0.318  44.148  -0.203   0.001   0.900   0.099
B V  3SE8H                169    0.057   8.792   0.181   0.001   0.900   0.099
B S  3SE8H                170    0.247  28.960   0.231   0.001   0.900   0.099
B W  3SE8H                171    0.060  14.550   0.095   0.018   0.846   0.136
E N  3SE8H                172    0.348  50.933   0.468   0.003   0.718   0.279
E S  3SE8H                173    0.523  61.342  -0.527   0.005   0.336   0.660
E G  3SE8H                174    0.589  46.370  -0.697   0.018   0.047   0.935
E A  3SE8H                175    0.556  61.282  -0.112   0.018   0.019   0.964
E L  3SE8H                176    0.542  99.277  -0.724   0.018   0.088   0.893
E T  3SE8H                177    0.541  75.078  -1.559   0.019   0.141   0.840
E S  3SE8H                178    0.562  65.902   0.134   0.004   0.138   0.858
E G  3SE8H                179    0.385  30.292  -0.640   0.005   0.336   0.660
B V  3SE8H                180    0.166  25.560  -0.356   0.004   0.616   0.381
E H  3SE8H                181    0.406  73.797   0.478   0.001   0.900   0.099
E T  3SE8H                182    0.317  44.037  -0.570   0.001   0.900   0.099
B F  3SE8H                183    0.218  43.712   1.134   0.002   0.816   0.182
E P  3SE8H                184    0.308  43.634  -0.603   0.004   0.514   0.481
B A  3SE8H                185    0.294  32.388  -0.946   0.003   0.718   0.279
B V  3SE8H                186    0.249  38.256  -1.376   0.002   0.816   0.182
E L  3SE8H                187    0.381  69.724  -0.103   0.002   0.816   0.182
E Q  3SE8H                188    0.429  76.548  -0.131   0.004   0.420   0.576
E S  3SE8H                189    0.731  85.708  -1.529   0.004   0.085   0.910
E S  3SE8H                190    0.782  91.604  -1.242   0.005   0.015   0.979
E G  3SE8H                191    0.468  36.792  -1.423   0.004   0.085   0.910
E L  3SE8H                192    0.382  70.017   0.926   0.004   0.616   0.381
B Y  3SE8H                193    0.142  30.303   0.056   0.001   0.900   0.099
B S  3SE8H                194    0.210  24.624  -0.192   0.001   0.900   0.099
B L  3SE8H                195    0.118  21.569  -0.546   0.001   0.959   0.040
B S  3SE8H                196    0.177  20.698  -0.351   0.001   0.959   0.040
B S  3SE8H                197    0.042   4.969  -1.153   0.001   0.959   0.040
B V  3SE8H                198    0.209  32.077   0.274   0.001   0.959   0.040
B V  3SE8H                199    0.051   7.808  -0.134   0.001   0.959   0.040
E T  3SE8H                200    0.278  38.628   0.846   0.001   0.900   0.099
B V  3SE8H                201    0.078  12.035   0.654   0.002   0.816   0.182
E P  3SE8H                202    0.357  50.616   1.315   0.022   0.552   0.426
E S  3SE8H                203    0.389  45.556  -0.089   0.021   0.279   0.699
E S  3SE8H                204    0.685  80.305  -0.638   0.021   0.279   0.699
E S  3SE8H                205    0.611  71.551   0.205   0.020   0.205   0.775
B L  3SE8H                206    0.191  34.935  -0.969   0.019   0.141   0.840
E G  3SE8H                207    0.698  54.917   0.740   0.005   0.015   0.979
E T  3SE8H                208    0.743 103.012   0.829   0.005   0.015   0.979
E Q  3SE8H                209    0.606 108.321   0.681   0.005   0.262   0.733
E T  3SE8H                210    0.518  71.833   1.199   0.002   0.816   0.182
B Y  3SE8H                211    0.081  17.267   0.514   0.001   0.959   0.040
E I  3SE8H                212    0.381  70.411   1.723   0.001   0.959   0.040
B C  3SE8H                213    0.027   3.847   0.031   0.001   0.959   0.040
E N  3SE8H                214    0.385  56.305   1.323   0.001   0.900   0.099
B V  3SE8H                215    0.064   9.898  -0.111   0.001   0.900   0.099
E N  3SE8H                216    0.548  80.286   1.018   0.001   0.900   0.099
B H  3SE8H                217    0.163  29.595   0.319   0.004   0.616   0.381
E K  3SE8H                218    0.662 136.235   0.709   0.005   0.045   0.951
E P  3SE8H                219    0.695  98.649  -1.289   0.005   0.015   0.979
E S  3SE8H                220    0.540  63.288  -0.295   0.005   0.045   0.951
E N  3SE8H                221    0.542  79.305  -0.152   0.004   0.085   0.910
E T  3SE8H                222    0.513  71.153   0.760   0.005   0.336   0.660
E K  3SE8H                223    0.527 108.404   1.749   0.003   0.718   0.279
B V  3SE8H                224    0.099  15.170   0.239   0.002   0.816   0.182
E D  3SE8H                225    0.654  94.198   0.635   0.001   0.900   0.099
E K  3SE8H                226    0.327  67.182   1.568   0.002   0.816   0.182
E K  3SE8H                227    0.594 122.206   1.169   0.003   0.718   0.279
B V  3SE8H                228    0.152  23.362   0.462   0.004   0.514   0.481
E E  3SE8H                229    0.571  99.789   0.721   0.005   0.262   0.733
E P  3SE8H                230    0.531  75.363  -0.168   0.004   0.085   0.910
E K  3SE8H                231    0.618 127.205  -0.247   0.018   0.088   0.893
E S  3SE8H                232    0.650  76.133   0.274   0.018   0.047   0.935
E C  3SE8H                233    0.535  75.184  -1.459   0.003   0.003   0.994

Explain the output.  Go back.
```

---
